# Identifying Macrophage-Related Genes in Ulcerative Colitis Using Weighted Coexpression Network Analysis and Machine Learning

**DOI:** 10.1155/2023/4373840

**Published:** 2023-10-25

**Authors:** Shaocheng Hong, Hongqian Wang, Shixin Chan, Jiayi Zhang, Bangjie Chen, Xiaohan Ma, Xi Chen

**Affiliations:** ^1^Department of Gastroenterology, The First Affiliated Hospital of Anhui Medical University, Hefei 230032, China; ^2^Anhui Provincial Key Laboratory of Digestive Diseases, Hefei, China; ^3^Department of General Surgery, The First Affiliated Hospital of Anhui Medical University, Hefei 230032, China; ^4^Department of Oncology, The First Affiliated Hospital of Anhui Medical University, Hefei 230032, China

## Abstract

Ulcerative colitis (UC) is an inflammatory bowel disease of unknown cause that typically affects the colon and rectum. Innate intestinal immunity, including macrophages, plays a significant role in the pathological development of UC. Using the CIBERSORT algorithm, we observed elevated levels of 22 types of immune cell infiltrates, as well as increased M1 and decreased M2 macrophages in UC compared to normal colonic mucosa. Weighted gene coexpression network analysis (WGCNA) was used to identify modules associated with macrophages and UC, resulting in the identification of 52 macrophage-related genes (MRGs) that were enriched in macrophages at single-cell resolution. Consensus clustering based on these 52 MRGs divided the integrated UC cohorts into three subtypes. Machine learning algorithms were used to identify ectonucleotide pyrophosphatase/phosphodiesterase 1 (ENPP1), sodium- and chloride-dependent neutral and basic amino acid transporter B(0+) (SLC6A14), and 3-hydroxy-3-methylglutaryl-CoA synthase 2 (HMGCS2) in the training set, and their diagnostic value was validated in independent validation sets. Gene set enrichment analysis (GSEA) and gene set variation analysis (GSVA) revealed the main biological effects, and that interleukin-17 was one of several signaling pathways enriched by the three genes. We also constructed a competitive endogenous RNA (CeRNA) network reflecting a potential posttranscriptional regulatory mechanism. Expression of diagnostic markers was validated in vivo and in biospecimens, and our immunohistochemistry (IHC) results confirmed that HMGCS2 gradually decreased during the transformation of UC to colorectal cancer. In conclusion, ENPP1, SLC6A14, and HMGCS2 are associated with macrophages and the progression of UC pathogenesis and have good diagnostic value for patients with UC.

## 1. Introduction

Ulcerative colitis (UC) is a complex autoimmune intestinal disease with unclear pathophysiological mechanisms that are associated with factors such as genetic background, intestinal flora, and mucosal immune dysregulation [1]. Its main feature is bloody diarrhea caused by ulceration of the mucosal layer. The global prevalence and incidence of UC have increased globally. For example, the prevalence of UC in the USA has reached 263 per 100,000 persons [2]. UC is a progressive disease that leads to intestinal stricture and dysfunction, and chronic UC can develop into colorectal cancer (CRC). The reported probability of patients with UC developing colitis-associated CRC (CAC) within >30 years is 18% [3, 4]. Although the emergence of antitumor necrosis factor-*α* (TNF-*α*) agents has significantly improved clinical treatment, the economic burden remains high [5]. Thus, new therapeutic targets and potential biological mechanisms require further exploration.

Macrophages play critical roles in chronic and acute inflammatory processes. In particular, an unbalanced M1/M2 macrophage ratio can arise due to overactivated M1-type cells that secrete enough proinflammatory factors to cause inflammatory storms. In contrast, M2-type cell populations reduce or remodel the microenvironment of chronic inflammation [6, 7]. Intestinal macrophages are essential in innate and classical antimicrobial immunity and regulate immune homeostasis [8]. A disrupted intestinal barrier in patients with UC leads to massive amounts of antigens entering the lamina propria, accompanied by a disrupted balance of immune tolerance [9, 10]. This, in turn, leads to local infiltration by numerous immune cells, such as proinflammatory macrophages and neutrophils. Proinflammatory macrophages exacerbate the dextran sulfate sodium (DSS)-induced intestinal inflammatory response in mice [11, 12].

The development of high-throughput technologies, transcriptome microarray data, and sequencing has led to the identification of biomarkers and underlying biological mechanisms of UC from the perspective of immune infiltration [13, 14]. However, few studies have targeted specific immune cells such as macrophages to identify relevant biomarkers in UC. Hence, we applied weighted gene coexpression network analysis (WGCNA) to screen for disease and macrophage-related gene modules. We obtained 52 macrophage-related genes (MRGs) after defining their intersections with differentially expressed genes (DEGs). We also used machine learning algorithms, including least absolute shrinkage and selection operator (LASSO) regression, Ranger, sliding window sequential forward feature selection (SWSFS), and support vector machine-recursive feature elimination (SVM-RFE) algorithms. We finally identified the target genes ectonucleotide pyrophosphatase/phosphodiesterase 1 (*ENPP1*), sodium- and chloride-dependent neutral and basic amino acid transporter B(0+) (*SLC6A14*), and 3-hydroxy-3-methylglutaryl-CoA synthase 2 (*HMGCS2*). Correlations between disease severity and each of these genes revealed the landscape of immune infiltration. Moreover, we revealed the involved regulatory molecular pathways using gene set enrichment analysis (GSEA) and gene set variation analysis (GSVA). The expression of diagnostic markers was validated in biospecimens and in vivo, respectively, and that dynamic HMGCS2 expression might be closely associated with inflammatory cancer transformation.

## 2. Materials and Methods

### 2.1. Data Acquisition and DEG Analysis

Microarray data downloaded from the Gene Expression Omnibus (GEO) database using the GEOquery R package comprised the GSE36807, GSE87466, GSE87473, GSE38713, GSE3629, GSE16879, GSE23597, GSE53306, GSE48959, GSE75214, and GSE13367 datasets [15–24]. All microarray datasets are UC samples and normal except GSE3629, which contains CRC and CAC samples. Gene expression data (transcripts per million (TPM)) for The Cancer Genome Atlas-colon adenocarcinoma (TCGA-COAD) cohort were downloaded from the Genomic *Data* Commons (GDC) portal. Following the guidance of previous studies, the single-cell sequencing (scRNA-seq) dataset GSE162335 was collected and analyzed [25].  Table 1 shows the accession numbers, platforms, and other details of the datasets. Because of the relatively large sample size of GSE87466, DEGs (|log2 fold change| > 1; false discovery rate (FDR) < 0.05) were obtained using the empirical Bayesian method in the limma R package [26].

### 2.2. Estimation of Immune Cell Infiltration and Correlations with Genes

We used the CIBERSORT algorithm of the multiomics Immuno-Oncology Biological Research (IOBR) package to compare the amounts of immune cell infiltration between UC and normal mucosal samples [27]. The perm parameter was set to 1,000 to obtain stable results. The CIBERSORT algorithm is a deconvolution method based on the LM22 reference matrix such that the sum of the calculated immune cell proportions for each sample is 1 [28]. Gene correlations with immune cells were determined using Spearman correlation analysis and further visualized using the ggplot2 package.

### 2.3. WGCNA Construction

We removed outliers, and then GSE36807 and GSE87466 samples were included in the WGCNA package to construct a gene expression similarity matrix that was subsequently transformed into an adjacency matrix by selecting the optimal soft threshold *β* to construct a scale-free network [29]. A dynamic tree-cutting algorithm was used to assign 10,000 included genes (ranked from largest to smallest according to median absolute deviation) to different modules comprising genes with similar expression profiles.

### 2.4. Batch Effect Correction and Consensus Clustering for 52 MRGs

We applied the ComBat algorithm of the sva R package to reduce the possibility of batch effects caused by nonbiotechnical bias between distinct datasets [30]. Based on the expression of 52 MRGs, an unsupervised clustering analysis was used to identify distinct MRGs modification patterns in UC patients and classify them for further investigation. This analysis was performed using the unsupervised clustering “Pam” method based on the Euclidean and Ward linkages, conducted through the use of the “ConsensusClusterPlus” R package and 1,000 replications to ensure stability of the classification [31].

### 2.5. Screening Genes and Building Four Classifiers Using Machine Learning

After a preliminary screening, the LASSO algorithm was first applied with penalized parameter adjustment by 10-fold cross-validation to select candidate genes [32]. We then used a weighted random forest (wRF) to assess the impact of gene expression on patient disease status using the Ranger package in R. The variable importance scores (VIS) of the 52 MRGs obtained from the initial screening were estimated and ranked in descending order. The parameters were set as described. The most important genes were identified using SWSFS that individually incorporates RF models according to the VIS rank of a gene [30]. When the RF model with the lowest out-of-bundle (OBB) rate was filtered out, the top gene in each set was identified as a candidate gene. We also combined candidate gene filtration using the SVM-RFE algorithm with the e1071 package in R to focus on candidate genes [33, 34]. We then incorporated the screened diagnostic gene markers to construct four machine learning classifiers, including RF, SVM, extreme gradient boosting (XGB) [35], and general linear model (GLM) [36].

### 2.6. GSEA and GSVA Analyses and Construction of CeRNA Networks

We used the clusterProfiler package [37] for specific GSEA to investigate biological processes that might be influenced by individual genes in samples from patients with UC. The background dataset was derived from the Kyoto Encyclopedia of Genes and Genomes (KEGG). Potential biological functions can be investigated using GSEA based on ordered gene expression profiles in two biological states. The enrichment of gene sets in individual patient samples can be assessed using GSVA [38]. We evaluated differences in biological process terms between groups with high and low expression of individual genes using the GSVA package in R. We also downloaded h.all.v7.2.symbols from MsigDB [39] and a inflammation-related signature [40] (*Supplementary 1*) for GSVA. Fast gene set enrichment analysis (fGSEA) was performed according to the Gene Ontology Biological Process (GOBP) with fGSEA R package. miRNAs targeting the three diagnostic markers were predicted via TargetScan (http://www.targetscan.org/), miRanda (http://www.microrna.org/), and miRDB (http://www.mirdb.org/) [41–43]. The lncRNAs targeting miRNAs were screened by spongeScan (http://spongescan.rc.ufl.edu), and the competitive endogenous RNA (CeRNA) network was constructed by Cytoscape software [44].

### 2.7. Patient Samples

Fresh colon tissues were obtained from six patients with UC who were treated by colonoscopy and six with CRC at the First Affiliated Hospital of Anhui Medical University. Normal tissues were obtained from paracancerous tissues of patients with colon cancer (CC). All samples were coded according to local ethical guidelines (as specified in the Declaration of Helsinki), and written informed consent was obtained from all patients to participate in the study. The study was approved by the Clinical Research Ethics Committee of the First Affiliated Hospital of Anhui Medical University (PJ 2022-10-41).

### 2.8. Immunohistochemistry (IHC)

Colon tissues were immersed in 4% paraformaldehyde for 24 hr, embedded in paraffin, sectioned, then oven-dried at 60°C for 30 min. Subsequently, HMGCS2 immunohistochemically detected using a primary anti-HMGCS2 antibody (T510043; Abmart, Shanghai, China) and an antirabbit secondary antibody for 30 min at room temperature. The tissue sections were stained using a 3,3ʹ-diaminobenzidine color development kit (ZLI-9017; Zhongshan Golden Bridge Biotechnology, Beijing, China), and then the intensity of HMGCS2 staining was analyzed using IPP6.0 software.

### 2.9. Animal Models and Western Blotting

Animal experiments were approved by the Animal Ethics and Experimentation Committee of Anhui Medical University and conducted in accordance with the Guide for the Care and Use of Laboratory Animals. Colitis was induced in 8-week-old female C57BL/LJ mice by the daily administration of 3% DSS salt (MP Biomedicals, Santa Ana, CA, USA) for 7 days.

Total protein extracted from the tissue using RIPA buffer (Beyotime Biotechnology, Shanghai, China) was resolved by sodium dodecyl sulfate-polyacrylamide gel electrophoresis and transferred to polyvinylidene fluoride (PVDF) membranes. Nonspecific antigen binding on the PVDF membranes was blocked with 5% skimmed milk for 1 hr at room temperature. The membranes were incubated overnight at 4°C with anti-HMGCS2 antibody (T510043; Abmart) followed by horseradish peroxidase (HRP)-conjugated secondary antibody for 1 hr at room temperature. Protein bands were quantified using enhanced chemiluminescence (ECL). Antibodies against SLC6A14 and CD206 were from Abmart (Shanghai, China). Antibodies against CD86, iNOS, and ARG1 were obtained from Proteintech Group (Wuhan, China).

### 2.10. Statistical Analysis

All data were statistically analyzed using R version 4.1.2 and visualized using the ggpubr package. The normal and nonnormal distributions between two groups of variables were assessed using independent *t*-tests and Wilcoxon rank-sum tests, respectively. The results of comparisons among three or more groups were assessed by parametric one-way analysis of variance (ANOVA) and nonparametric Kruskal–Wallis tests. Correlations between groups were assessed based on the normality of the data using Spearman or Pearson correlation tests. We determined the diagnostic value of individual genes by calculating areas under ROC curves (AUC) using the pROC package [45]. Values with *P* < 0.05 were considered significant, and data are represented as means ± standard deviation (SD).

## 3. Results

### 3.1. Immune Infiltration Landscape in UC

**Figure 1:**
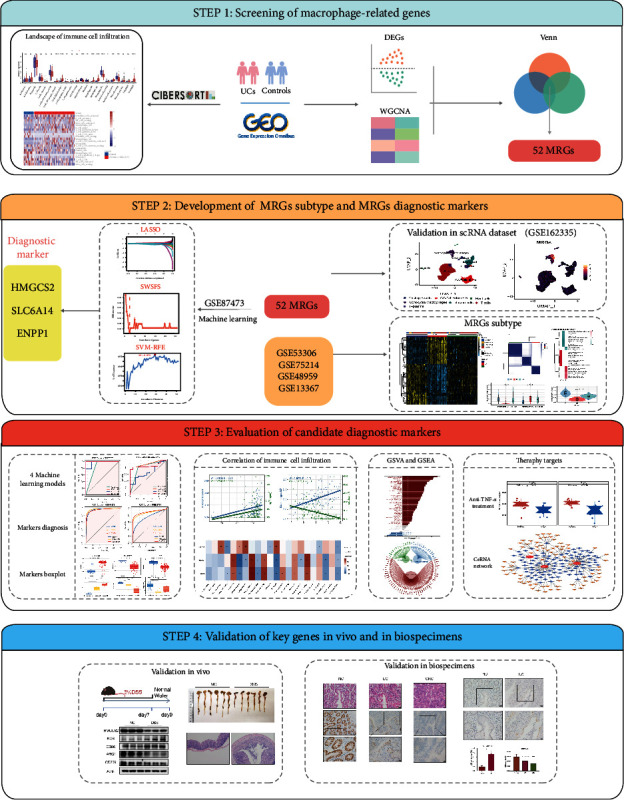
The overall flowchart of this research.

**Figure 2:**
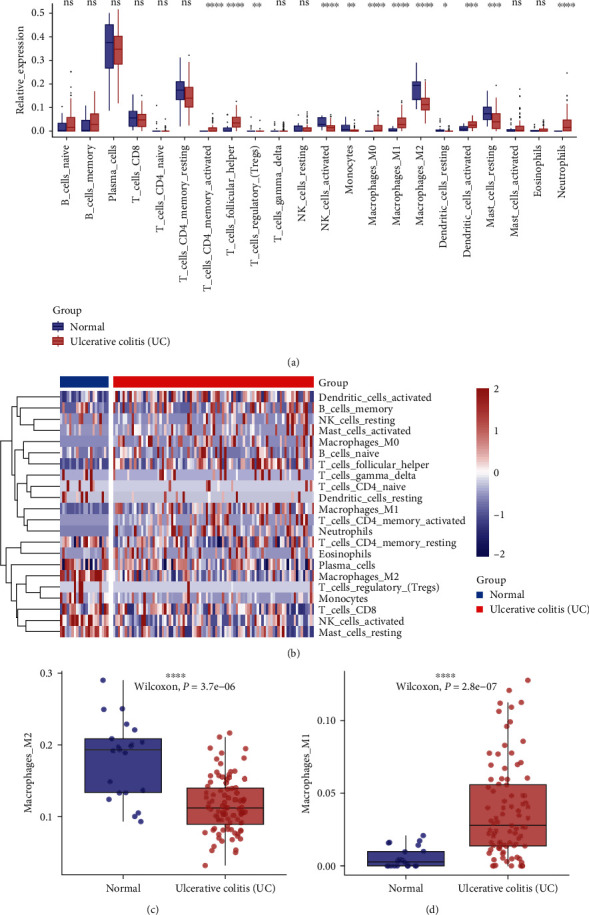
Assessment of the immune infiltration landscape of UC and control samples in the GSE87466 dataset. (a) Box plot shows infiltration of 22 immune cells in control and UC samples. (b) Heat map of 22 types of infiltrative immune cells. (c and d) Boxplot shows infiltration of M1 and M2 macrophages.

Figure 1 shows a flowchart of the study. Because chronic inflammatory UC is associated with dysregulated immune infiltration, we used the CIBERSORT algorithm to evaluate the infiltration of 22 types of immune cells in normal and UC tissues from the GSE87466 dataset based on the LM22 reference matrix. We found significantly enriched M0 and M1 macrophages, as well as neutrophils in the UC tissues and mainly enriched M2 macrophages and activated natural killer (NK) cells in the normal tissues (Figure 2(a)–2(d)). It follows that immune cells, in particular macrophages, could play a key role in UC's development.

### 3.2. WGCNA Construction and DEG Analysis

**Figure 3:**
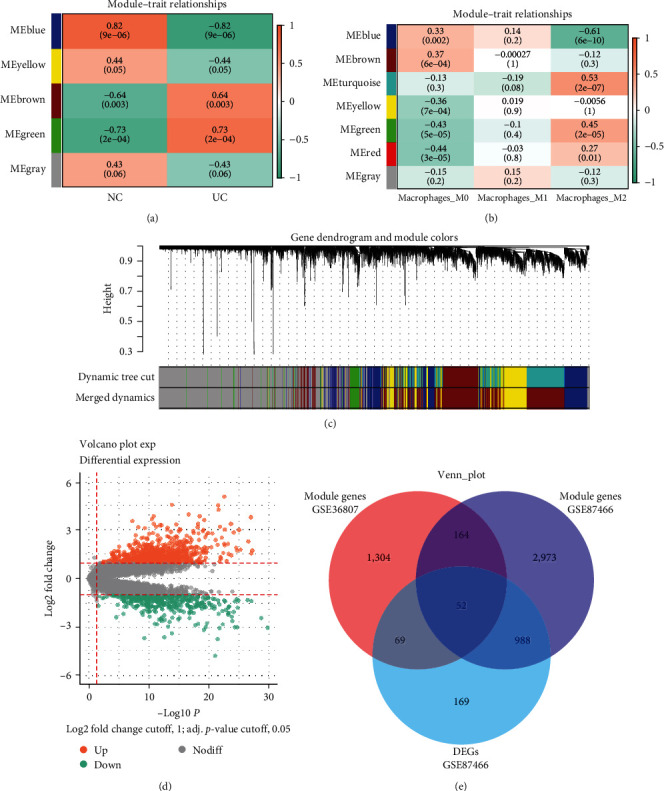
WGCNA construction and DEG analysis. (a and b) Correlations of module eigengenes with macrophages and UC traits. Cellular data are presented as Pearson coefficients (rho; *ρ*). (c) Hierarchical cluster trees constructed by dynamic hybrid cutting, in which leaves represent genes and branches represent coexpression modules. (d) Volcano plots of DEGs in UC and control tissues from GSE36807. (e) Venn plot shows 52 intersecting DEGs and genes determined by WGCNA.

In order to gain insights into the differences between UC samples and normal control samples, we utilized the GSE36807 dataset for WGCNA. Cluster analyses of samples in the GSE36807 dataset excluded two abnormal samples (GSM901353, GSM901339), and the remaining 20 samples were subsequently analyzed (*Supplementary 2*). We set *β* = 27 (scale-free *R*^2^ = 0.85) as the soft threshold to construct a scale-free network (*Supplementary 2*). We identified UC-related modules and merged those that were similar using the dynamic cut-tree method to obtain five modules (Figure 3(c)). The MEblue (cor = −0.82, *P* = 9e-06) and MEgreen (cor = 0.73, *P* = 2e-04) modules were most relevant to UC (Figure 3(a)). The results depicted as scatter plots of the gene significance of the relevant modules were similar (*Supplementary 2*).

To identify specific genes associated with macrophage infiltration in the UC pathological process, we expanded our analysis by including 85 UC samples from the GSE87466 dataset. We followed a similar process for scale-free network construction, applying a soft threshold *β* value of 16 (*Supplementary 2*). Heat maps of trait correlations showed that blue, green, and turquoise modules closely correlated with macrophage infiltration in UC (Figure 3(b)), implying that they are associated with the development of inflammation in UC. *Supplementary 2* shows scatter plots of module relevance *versus* gene significance.

A comparison of UC and normal samples from GSE87466 revealed 1,287 DEGs, of which 415 and 872 were downregulated and upregulated, respectively (Figure 3(d)). The Venn diagram, as shown in Figure 3(e), shows that the key module genes determined by WGCNA intersected with these DEGs and resulted in 52 candidate genes (*Supplementary 1*).

### 3.3. Validation of MRGs at Single-Cell Resolution and Identification of Three MRGs Subtypes

**Figure 4:**
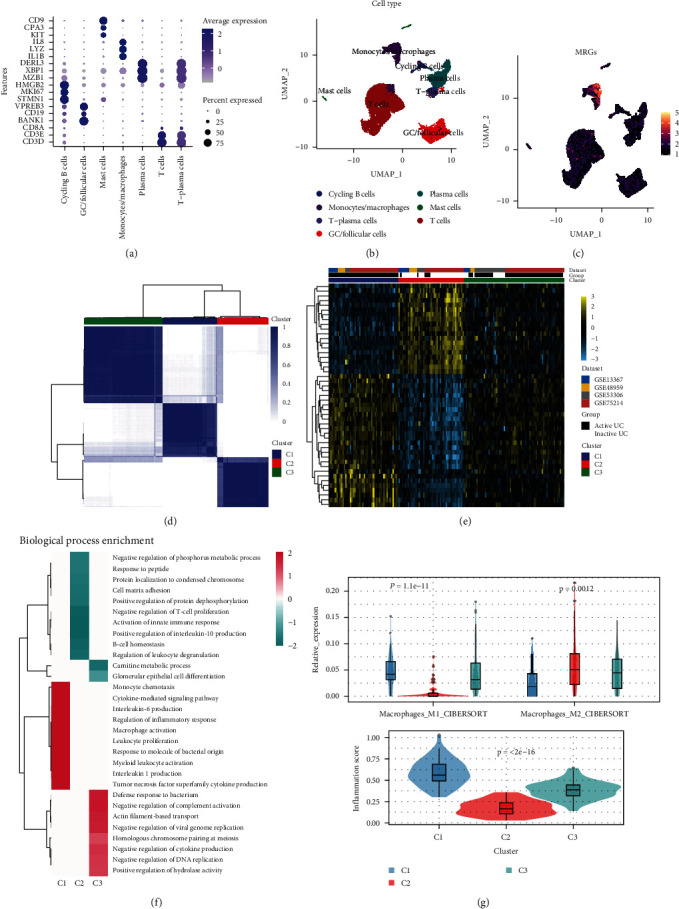
Validation of ARGs at single-cell resolution and stratification of integrated UC cohorts. Dot plot (a) of cell type marker genes and UMAP (b) of major immune cell clusters. (c) UMAP of MRGs signature enriched in major cell clusters. (d) Heat map depicting consensus clustering solution (*k* = 3) for macrophage-related genes in UC samples (e) Unsupervised clustering of the 52 ARGs divided the UC patients in the integrated cohort into three groups. (f) The main 10 pathways in the three subtypes were significantly activated (red) or inhibited (green) according to fGSEA. (g) Violin plots (box plots) of macrophage infiltration levels and inflammation scores for the three subtypes.

We identified seven major subpopulations using cell-specific markers, namely T cells, plasma cells, circulating B cells, germinal center/follicular cells, mast cells, T-plasma cells, and monocytes/macrophages (Figures 4(a) and 4(b)). In line with our expectations, MRGs were mainly enriched in monocytes/macrophages (Figure 4(c)).

Four GEO datasets (GSE53306, GSE48959, GSE75214, and GSE13367) were enrolled into one metacohort. Principal component analysis (PCA) confirmed a reduction in between-datasets batch effects after correction (*Supplementary 2*). We then performed a consensus cluster analysis to investigate the relationship between these MRGs and UC subtypes. Based on CDF values, we classified 155 UC patients into three clusters (*k* = 3, Figure 4(d); *Supplementary 2*) and we found that all patients with C1 were active UC and had higher M1 macrophage infiltration and inflammation scores (Figures 4(e) and 4(g)). In addition, the C1 subtype was predominantly enriched for biological processes such as myeloid leukocyte activation and regulation of monocyte chemotaxis (Figure 4(f)). Besides, based on this integrated cohort, we also found that active and inactive UC were significantly different (*Supplementary 2*). Inactive UC had higher M2 macrophage infiltration and lower inflammation scores (*Supplementary 2*). Differential expression analysis between the two groups yielded 265 DEGs (|Log2 fold change| > 1; FDR < 0.05), which were mainly enriched in biological processes related to inflammatory pathways (*Supplementary 2*). We further used these DEGs to construct a protein–protein interaction network (*Supplementary 2*) based on protein interactions in the STRING database [46].

### 3.4. Screening Biomarkers for UC Using Machine Learning Algorithms

**Figure 5:**
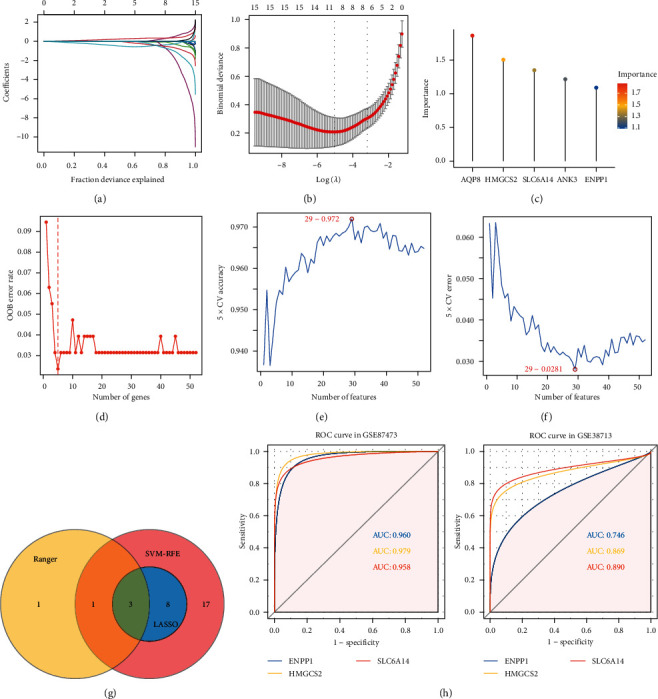
Identification of potential biomarkers for UC based on machine learning algorithms. (a and b) LASSO regression screening variables. (c and d) Ranger provided variable importance scores (VIS) for five genes only in patients with UC. Out-of-bag (OOB) error rate of these genes in the model together with genes included individually based on their VIS ranks. (e and f) Optimal error rate and accuracy of SVM model based on 29 characteristic genes. (g) Venn diagram shows overlapping genes in LASSO, SVM, and SWSFS. (h) ROC curves of *SLC6A14*, *HMGCS2*, and *ENPP1* in tissues from GSE87473 and GSE38713.

We further reduced the dimensionality and filtered the 52 candidate genes using three machine learning algorithms in GSE87473. The first was LASSO regression based on UC and normal samples. The results showed that the contraction of genes tended to stabilize. Binomial deviance was minimized when the following genes were included: tissue factor pathway inhibitor 2 (*TFPI2*), cadherin 3 (*CDH3*), *SLC6A14*, homeobox A3 (*HOXA3*), phosphodiesterase 6A (*PDE6A*), high-mobility group AT-hook 2 (*HMGA2*), complement factor B (*CFB*), V-Set and transmembrane domain containing 2A (*VSTM2A*), interferon stimulated exonuclease *gene* 20 (*ISG20*), *HMGCS2*, and *ENPP1*, and the optimal *λ* was 0.0064 (Figures 5(a) and 5(b)).

Moreover, the SWSFS algorithm with random seeds set to 5,555 identified a model containing the top five genes such as aquaporin 8 (*AQP8*), *HMGCS2*, *SLC6A14*, ankyrin 3 (*ANK3*), and *ENPP1* (Figures 5(c) and 5(d)). Meanwhile, the SVM-RFE algorithm identified 29 signature genes with an optimum error and accuracy rate of 0.0281 and 0.972, respectively (Figures 5(e) and 5(f)). Combining with the above findings, *SLC6A14*, *HMGCS2*, and *ENPP1* were selected as crucial genes (Figure 5(g)).

### 3.5. Evaluation of Expression and Diagnostic Value of Potential Macrophage-Related Genes

**Figure 6:**
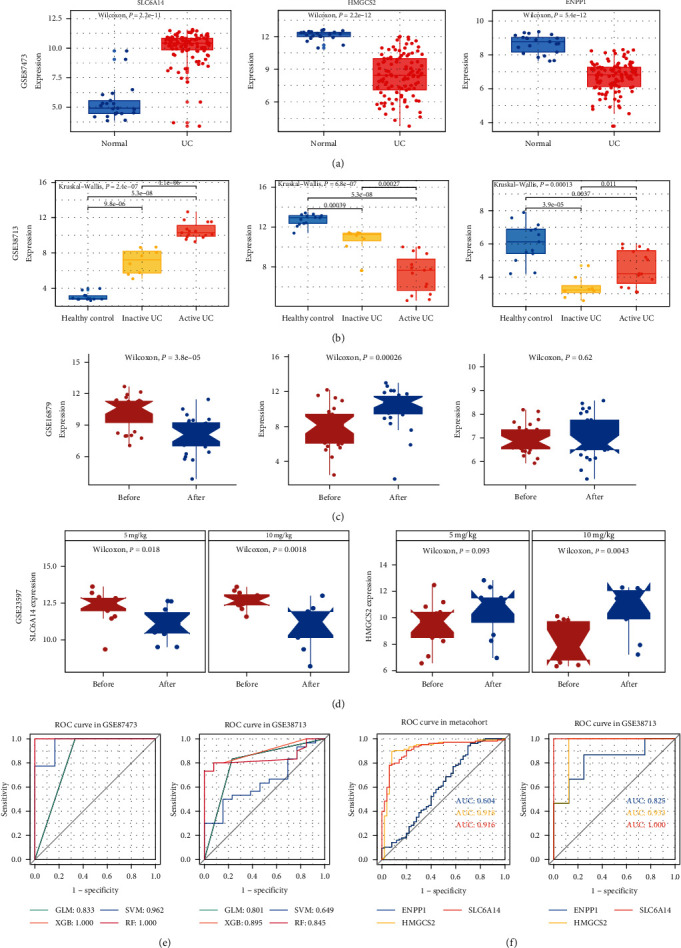
Diagnostic value and expression of three candidate genes. Expression of *SLC6A14*, *HMGCS2*, and *ENPP1* in UC and control tissues in GSE87473 (a) and in tissues from GSE38713 (b). Expression of candidate diagnostic markers before and after treatment in infliximab treatment responders in GSE16879 (c) and GSE23597 (d). (e) ROC curves for four machine learning classifiers based on three candidate diagnostic markers in the training set GSE87473 and test set GSE38713. (f) ROC curves for identifying activate UC based on three candidate diagnostic markers in the metacohort and GSE38713.

We analyzed receiver operating characteristics (ROC) curves to determine the predictive value of *SLC6A14*, *HMGCS2*, and *ENPP1*. The areas under the ROC curves (AUCs) were 0.958, 0.979, and 0.960, respectively, for GSE87473. The GSE38713 validation set revealed AUCs of 0.890, 0.869, and 0.746 for *SLC6A14*, *HMGCS2*, and *ENPP1*, respectively (Figure 5(h)). We then evaluated their expression and found that *SLC6A14* was upregulated in both the training and validation cohorts compared to that in the control, whereas both *HMGCS2* and *ENPP1* were downregulated (Figure 6(a); *Supplementary 2*). In addition, the expression of *SLC6A14* and *HMGCS2* varied with the severity of UC (Figure 6(b)), and, interestingly, the expression levels of these genes also changed in infliximab treatment responders (Figures 6(c) and 6(d)), indicating that they are involved in the development of UC. Based on these three diagnostic makers using four machine learning algorithms (SVM, RF, XGB, and GLM) to construct diagnostic classifiers, we found that all three classifiers, except SVM, had good diagnostic value in both the training and validation cohorts (Figure 6(e)). Additionally, we evaluated the diagnostic value of three candidate diagnostic markers in an integrated cohort and in individual cohorts to identify the active phase of UC (Figure 6(f); *Supplementary 2*).

### 3.6. Immune Infiltration Correlation

**Figure 7:**
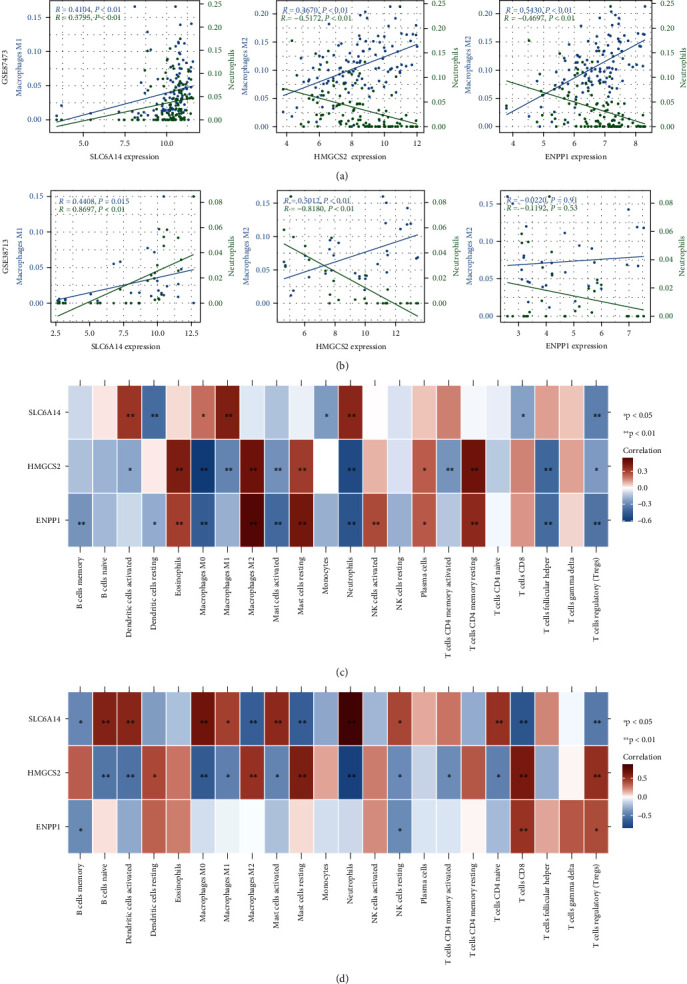
Correlation analysis to assess immune infiltration. (a and b) Scatter plots of correlations between *SLC6A14*, *HMGCS2*, and *ENPP1* expression and macrophages and neutrophils in GSE87473 and GSE38713. (c and d) Heat map shows correlations among *SLC6A14*, *HMGCS2*, and *ENPP1* and 22 types of immune cells in GSE87473 and GSE38713 (data are shown as Spearman *ρ* and *P*-values).

We further confirmed the potential biological processes of *SLC6A14*, *HMGCS2*, and *ENPP1* by analyzing correlations with immune infiltrative cells. The expression of *SLC6A14* significantly correlated with scores estimated for neutrophils and M1 macrophages in GSE87473 and GSE38713, respectively. The expression of *HMGCS2* correlated significantly and positively with M2 macrophages and negatively with neutrophils. Although the performance of *ENPP1* and *HMGCS2* was similar in the GSE87473 cohort, it was not validated in GSE38713 (Figures 7(a) and 7(b)). The heat map, as shown in Figures 7(c) and 7(d), shows that these genes correlated with 22 types of infiltrative immune cells identified by CIBERSORT.

### 3.7. Biological Process Enrichment and CeRNA Networks Construction

**Figure 8:**
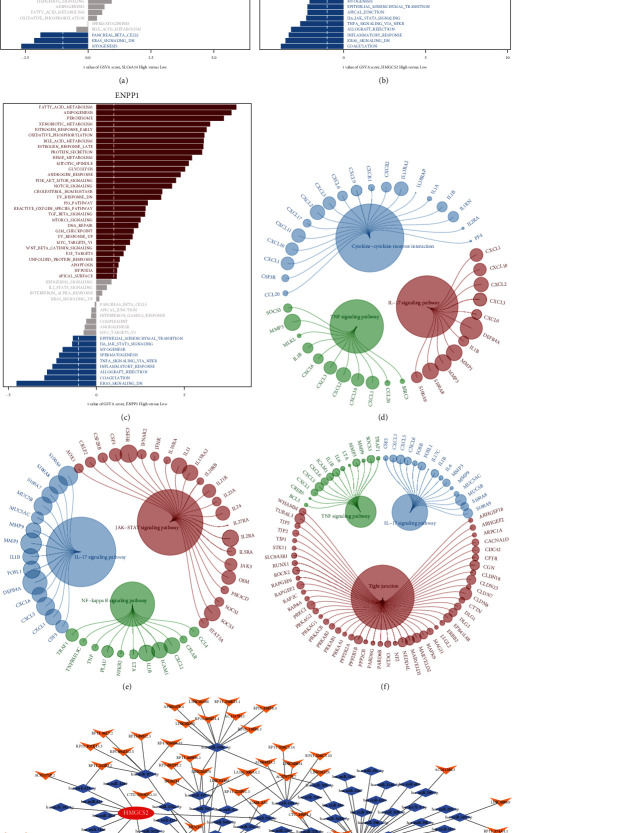
GSVA and GSEA enrichment analysis and CeRNA networks. (a–c) Bar plots show GSVA scores of hallmark signaling in tissues with high and low gene expression. Red and blue significantly upregulated and downregulated signaling pathways, respectively. Gray, nonsignificant signaling. (d–f) The enrichment of three genes analyzed using the KEGG pathway of GSEA. (g) CeRNA networks of HMGCS2, SLC6A14, and ENPP1.

We compared the GSVA scores of hallmark signaling pathways between UC with high and low expression of these genes (Figure 8(a)–8(c); *Supplementary 1*). The significantly enriched inflammation-related pathways in the group with abundant SLC6A14 expression were INFLAMMATORY_RESPONSE, IL6_JAK_STAT3_SIGNALING, and TNFA_SIGNALING_VIA_NFKB. Meanwhile, these pathways were substantially enriched in groups with low *HMGCS2* and *ENPP1* expression. *SLC6A14*, *HMGCS2*, and *ENPP1* were also enriched in several inflammation-related pathways such as interleukin 17 (IL-17), nuclear factor-kappa B (NF-*κ*B), and TNF-*α* signaling in the background KEGG dataset for GSEA (Figure 8(d)–8(f)). A CeRNA network consisting of three mRNAs, 95 miRNAs, and 74 lncRNAs was constructed based on predicted miRNA–mRNA and miRNA–lncRNA interactions (Figure 8(g); *Supplementary 1*).

### 3.8. HMGCS2 Correlates with Inflammatory Cancer Transformation

**Figure 9:**
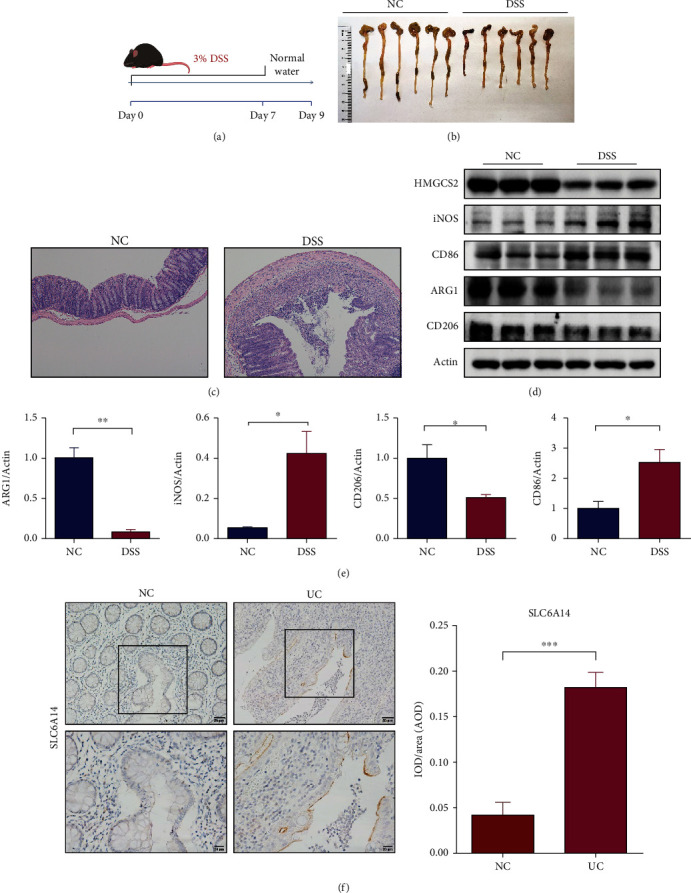
Validation in a 3% DSS-induced acute colitis mouse model and biospecimens. (a) Schematic of the 3% DSS-induced acute ulcerative colitis model. (b) Determination of the length of the colon in mice. (c) Representative images of H&E-stained colonic tissue at day 9 of colitis induction. (d and e) The relative expression levels of HMGCS2, ARG1, CD86, iNOS, and CD206 were measured by western blotting in the colonic tissues from DSS-induced colitis model and control mice. (f) Expression of SLC6A14 determined by IHC in six pairs of normal and UC colon tissues ( ^*∗*^*p* < 0.05,  ^*∗∗*^*p* < 0.01,  ^*∗∗∗*^*p* < 0.001 in *t*-test).

**Figure 10:**
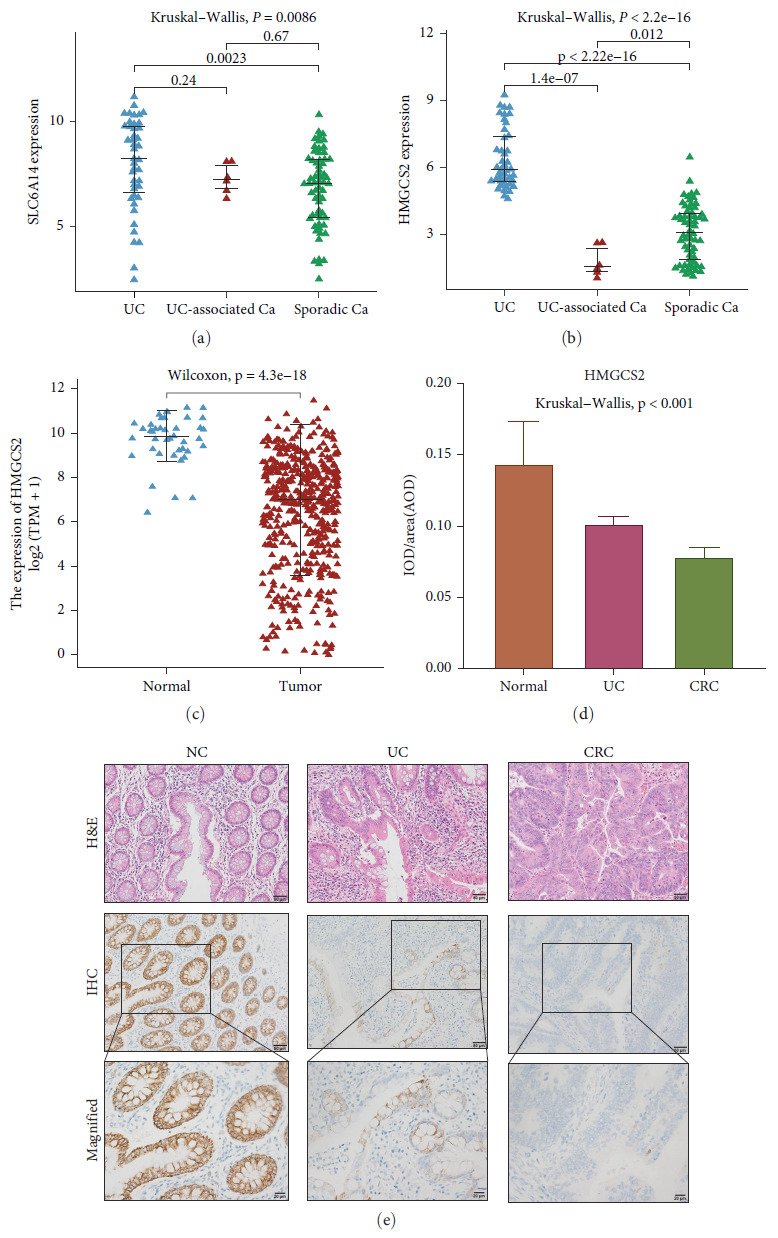
Association between HMGCS2 and inflammatory cancer transformation. Expression of SLC6A14 (a) and HMGCS2 (b) in UC, CAC, and sporadic CC. (c) Expression of HMGCS2 in CC and normal tissues in TCGA-COAD cohort. (d and e) Expression of HMGCS2 determined by IHC in six pairs of normal, UC, and CRC colon tissues.

To further validate the key diagnostic genes, we applied a mouse model of DSS-induced acute colitis (Figure 9(a)–9(c)). Western blotting revealed reduced expression of HMGCS2, ARG1, and CD206 and increased expression of iNOS and CD86 in colon tissues from the mouse models of DSS-induced colitis, further validating these findings (Figures 9(d) and 9(e)). In addition to this, we found that SLC6A14 expression was elevated in colonic tissue from UC patients compared to control tissue (Figure 9(f)). The expression of SLC6A14 and HMGCS2 was analyzed in UC, UC-associated CRC, and sporadic CC (Figures 10(a) and 10(b)). Compared to that in UC tissues, significantly less HMGCS2 was expressed in colorectal tumors. Besides, HMGCS2 expression was also reduced in CC tissues relative to normal tissues in the COAD cohort of the TCGA database (Figure 10(c)). However, our analysis through the public repositories, cBioPortal (https://www.cbioportal.org/) found no significant differences in the methylation status of HMGCS2 and its expression at different staging of CRC (*Supplementary 2*). Moreover, IHC revealed that HMGCS2 expression progressively decreased in normal, UC, and CRC tissues (Figures 10(d) and 10(e)).

## 4. Discussion

Intestinal immune dysregulation, including macrophages, neutrophils, and other immune cell infiltrates, is an important pathological feature of UC [47]. Macrophages have been extensively studied in UC due to their high plasticity and intestinal heterogeneity [48, 49]. We compared the normal immune landscape with that of patients with UC using the CIBERSORT algorithm. Consistent with previous results, we identified significantly more infiltrative M1 macrophages, neutrophils, and activated DC cells and significantly fewer infiltrative M2 macrophages in the patients with UC than those without UC. We identified 52 MRGs using WGCNA and DEG analysis.

MRGs were subsequently validated at single-cell resolution to be predominantly enriched in monocytes/macrophages. Further, we first identified three subtypes of MRGs for UC and found that patients with UC in subtype C1 were all active while subtype C2 had mainly inactive UC. Consistent with this, subtype C1 had the highest inflammation score and M1 macrophage infiltration of the three subtypes, while subtype C2 had the lowest inflammation score and highest M2 macrophage infiltration.

The combination of the LASSO, SWSFS, and SVM-RFE machine learning algorithms for variable screening led to targeting the candidate markers, *SLC6A14*, *HMGCS2*, and *ENPP1*. Subsequent correlations between immune cells and gene expression were validated in external datasets.

Among these macrophage-associated genes, *SLC6A14* was elevated in UC, compared to that in control tissues, whereas *HMGCS2* and *ENPP1* expressions were decreased. Yanai et al. [50] also found significantly elevated *SLC6A14* expression in tissues from patients who developed pouchitis after restorative proctocolectomy. In addition, inflammation-related CRC is prevented in *SLC6A14*-deficient mice [51]. Based on the proteomic platform, *HMGCS2* protein expression was reduced in tissues from patients with UC compared to that in healthy controls [52]. Besides, HMGCS2 acts as a rate-limiting enzyme for ketogenesis to alleviate TNF-*α*-induced inflammation in intestinal epithelial cells [53]. However, the relationship between ENPP1 and UC has essentially remained obscure.

Macrophages are categorized as being classically (M1) or alternatively (M2) activated depending on their pro- or anti-inflammatory phenotypes [7]. In the context of UC pathogenesis, chemokines induce monocytes to travel to regions of colonic inflammation where they differentiate into the M1 phenotype that promotes inflammation. However, tissue-resident M2-like macrophages play a tissue-repairing, anti-inflammatory role in colitis [54, 55]. Our results indicated that *SLC6A14* and HMGCS2 correlate positively with M1 and M2 macrophages, respectively, and with neutrophils in UC. The amount of neutrophil infiltration in colonic tissues increases in parallel with UC progression [56]. In addition, evidence suggests that neutrophils accumulate around active ulcers in UC and that chemokine (IL-8) signals result in neutrophil degranulation followed by the release of myeloperoxidase (MPO) that mediates oxidative stress to produce cytotoxic reactive oxygen species [57, 58]. Various evidence now suggests that neutrophils and inflammation-induced macrophages are involved in the development of CAC. For instance, the proportions (%) of CD68+ macrophages sequentially increase during the transition of normal mucosa into inflammatory hyperplasia and cancer in azoxymethane (AOM)-/DSS-induced model mice [59]. Depleting macrophages with clodronate liposomes twice weekly before the final DSS cycle (week 7) in DSS/AOM mouse models reduces the number and size of colon tumors [60]. In addition, neutrophil infiltration is significantly increased in the mouse models of DSS-/AOM-induced CAC [61, 62]. We also found using public microarray data that HMGCS2 expression progressively decreased from normal to UC to CRC and reconfirmed this using IHC. Coincidentally, *HMGCS2* knockdown exacerbates the macrophage-activated inflammatory response in acute pancreatitis [63]. Therefore, we speculate that HMGCS2 also mitigates the development of inflammation in UC and even participates in the process of inflammatory cancer transformation by limiting inflammatory macrophage infiltration.

Our findings revealed that all three genes were enriched in the IL-17 and TNF-*α* signaling pathways. The proinflammatory cytokine IL-17 is associated with the development and progression of UC [64]. Although IL17 is secreted by various cells, a T-cell lineage producing IL17 that is distinct from Th1 and Th2 has been identified in UC [65, 66]. The key cytokine TNF-*α* is elevated in patients with UC and can significantly reduce intestinal barrier resistance, leading to a defective intestinal barrier. Anti-TNF-*α* therapy is also effective for patients with UC, especially those with moderate-to-severe disease who cannot tolerate conventional drug therapy [67]. Our results also showed significant changes in HMGCS2 and SLC6A14 in anti-TNF-*α* treatment responders before and after treatment. These two markers also correlate with the level of activity in UC. Previous study has summarized markers related to UC activity [68], while our analysis results differs from previous studies, possibly due to the limitations of the datasets and sample size. Further, we constructed a CeRNA network based on three diagnostic marker genes in order to identify potential posttranscriptional regulatory mechanisms. All these findings imply that the macrophage-related genes determined from our screen could be promising therapeutic targets for UC.

However, our study has several limitations. The amounts of clinical information and samples were limited. Although we classified UC patients into three groups based on 52 MRG genes, this classification cannot currently be well related to the clinical classification of UC based on this result alone. Moreover, our findings of HMGCS2 expression and its correlation with clinical features require validation in a larger patient cohort. The number of experiments in vivo was insufficient for us to validate the potential pathogenesis of HMGCS2 in UC and CAC.

## 5. Conclusions

In summary, we identified three MRGs subtypes for UC and the macrophage-associated genes *SLC6A14*, *HMGCS2*, and *ENPP1* and further analyzed their involvement in inflammation-related pathways. Expression of HMGCS2 and macrophage polarization markers was verified in DSS-induced colitis mice model. We also validated the high expression of SLC6A14 in UC in biospecimens by IHC and associated HMGCS2 with the process of inflammatory cancer transformation, which also reflects that these genes could be a potential therapeutic target for UC.

## Figures and Tables

**Table 1:** Basic information of datasets.

Accession number/source	Platform	Number of patients	Tissues
GEO:GSE36807	GPL570	22	7 controls and 15 UC
GEO:GSE87466	GPL13158	108	21 controls and 87 UC
GEO:GSE87473	GPL13158	127	21 controls and 106 UC
GEO:GSE38713	GPL570	43	13 controls and 30 UC
GEO:GSE3629	GPL570	121	53 UC, 6 CAC, and 62 CRC
GEO:GSE16879	GPL570	28	39 CD and 16 UC
GEO:GSE23597	GPL570	26	45 UC
GEO:GSE53306	GPL14951	24	12 controls and 28 UC
GEO:GSE48959	GPL6244	21	8 controls and 13 UC
GEO:GSE75214	GPL6244	108	11 controls and 97 UC
GEO:GSE13367	GPL570	27	10 controls and 17 UC
GEO:GSE162335	Illumina HiSeq 4000	11	11 UC
TCGA:COAD	Illumina RNAseq	521	41 controls and 480 CRC

## Data Availability

Datasets can be found in online repositories for this study. The names of the repository/repositories and accession number(s) can be found below: https://www.ncbi.nlm.nih.gov/ and https://portal.gdc.cancer.gov/. R scripts for analyzing data are available on reasonable request.

## Supplementary Materials

Supplementary 1 Table S1: 52 macropage-related genes. Table S2: inflammatory genes. Table S3: GSVA and GSEA output. Table S4: CeRNA networks.

Supplementary 2 Figure S1: integration of datasets batch correction and consensus clustering matrixes of UC patients. Figure S2: soft thresholding power and scale-free network validation in GSE36807 and GSE87466. Figure S3: differentially expressed genes between active and quiescent UC. Figure S4: diagnostic value of candidate diagnostic markers in identifying active UC. Figure S5: expression of candidate markers in the UC cohort of the GSE38713 dataset and in the TCGA CRC cohort with different tumor stages.
