# Supplementary figures and images for: Identifying Macrophage-Related Genes in Ulcerative Colitis Using Weighted Coexpression Network Analysis and Machine Learning
Source: Mediators Inflamm. 2023 Oct 25;2023:4373840. doi: 10.1155/2023/4373840 (PMC11023725; doi:10.1155/2023/4373840)

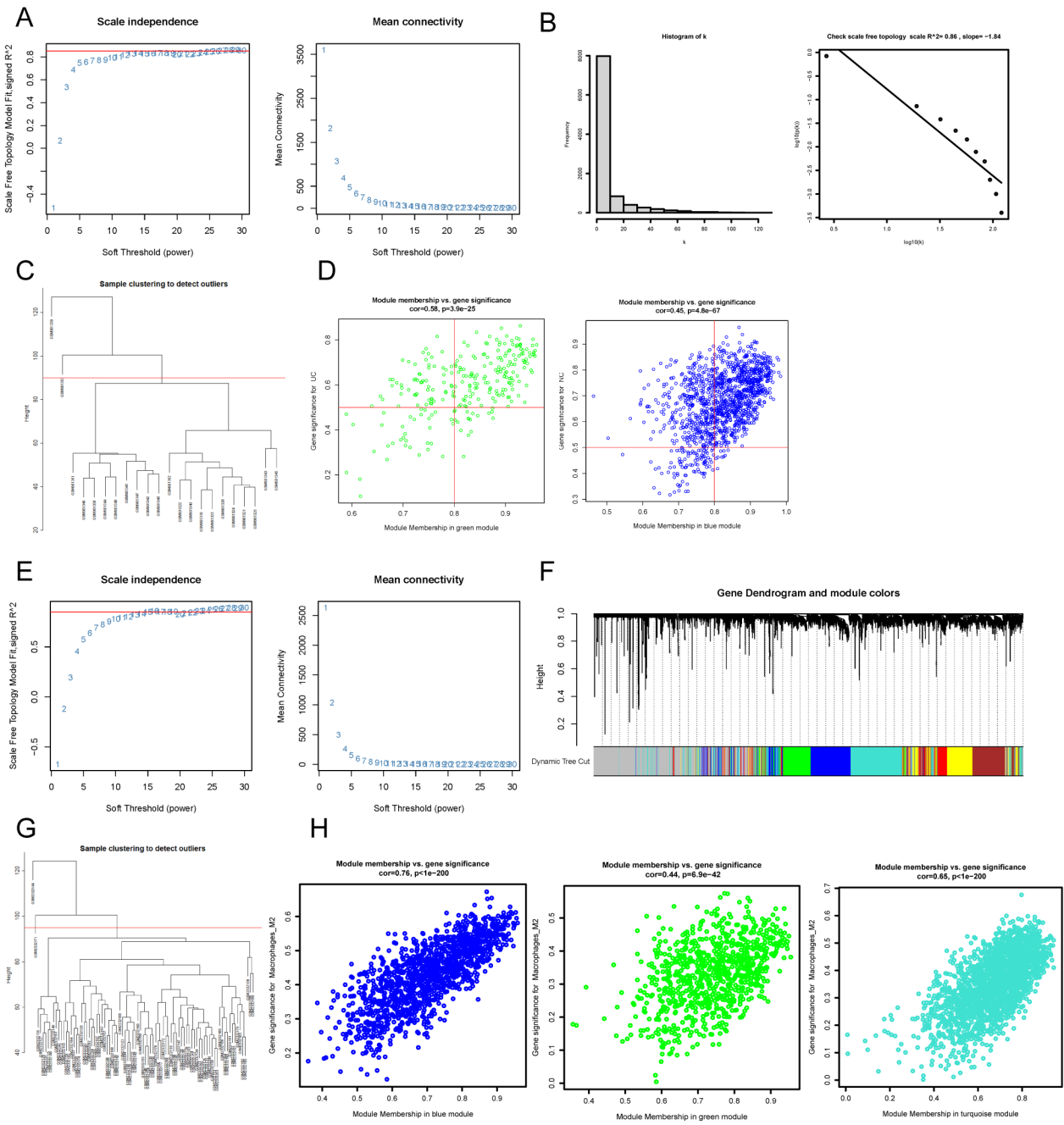

FIG-S1

A

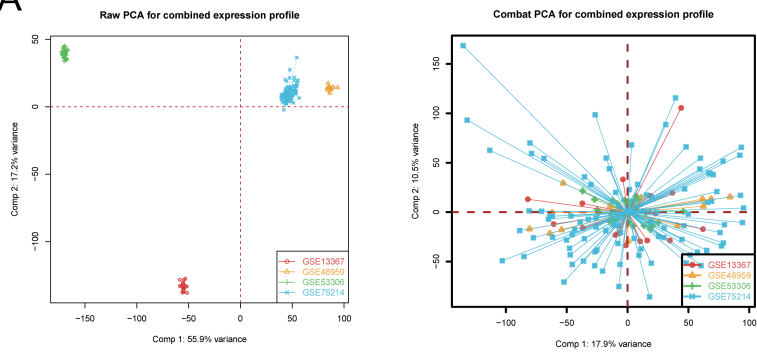

B

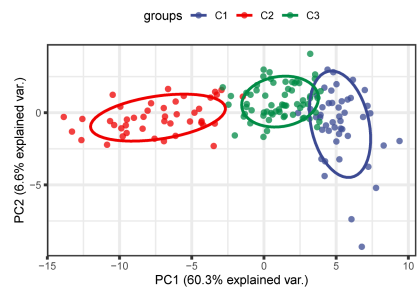

C

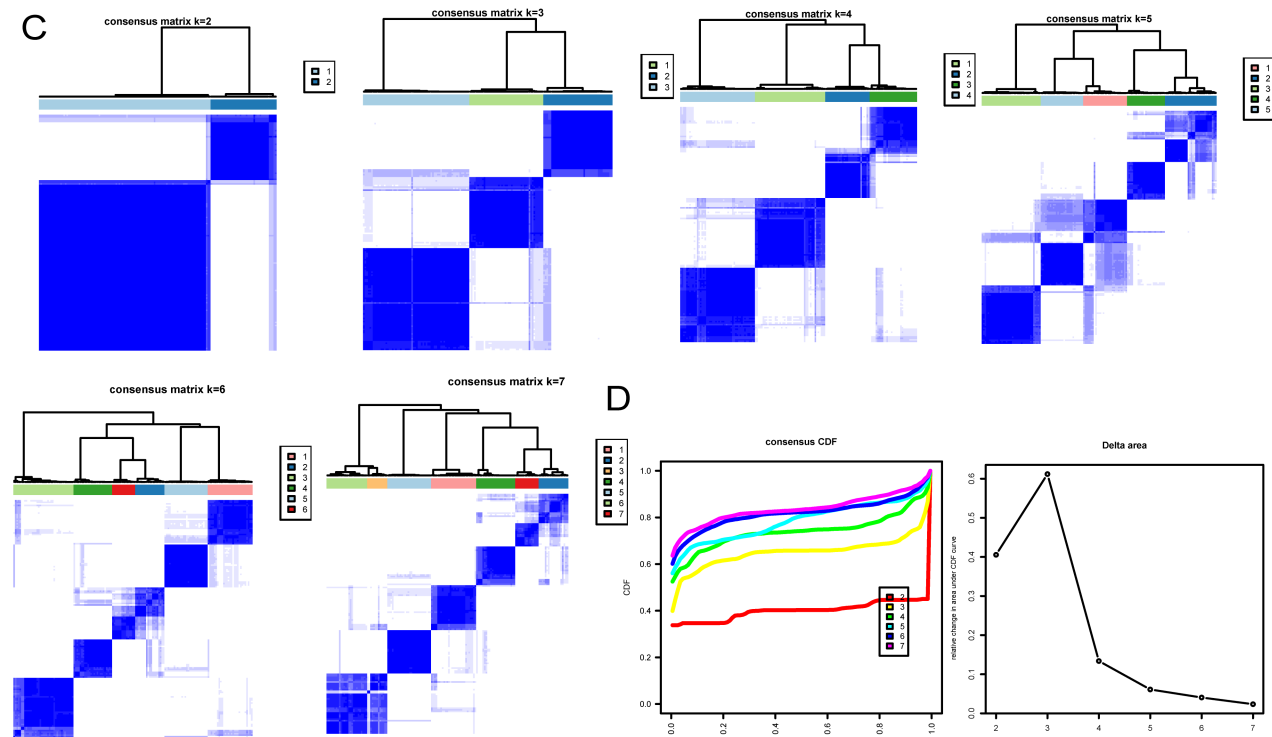

D

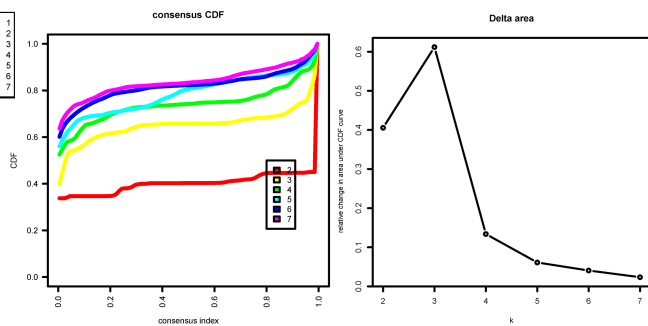

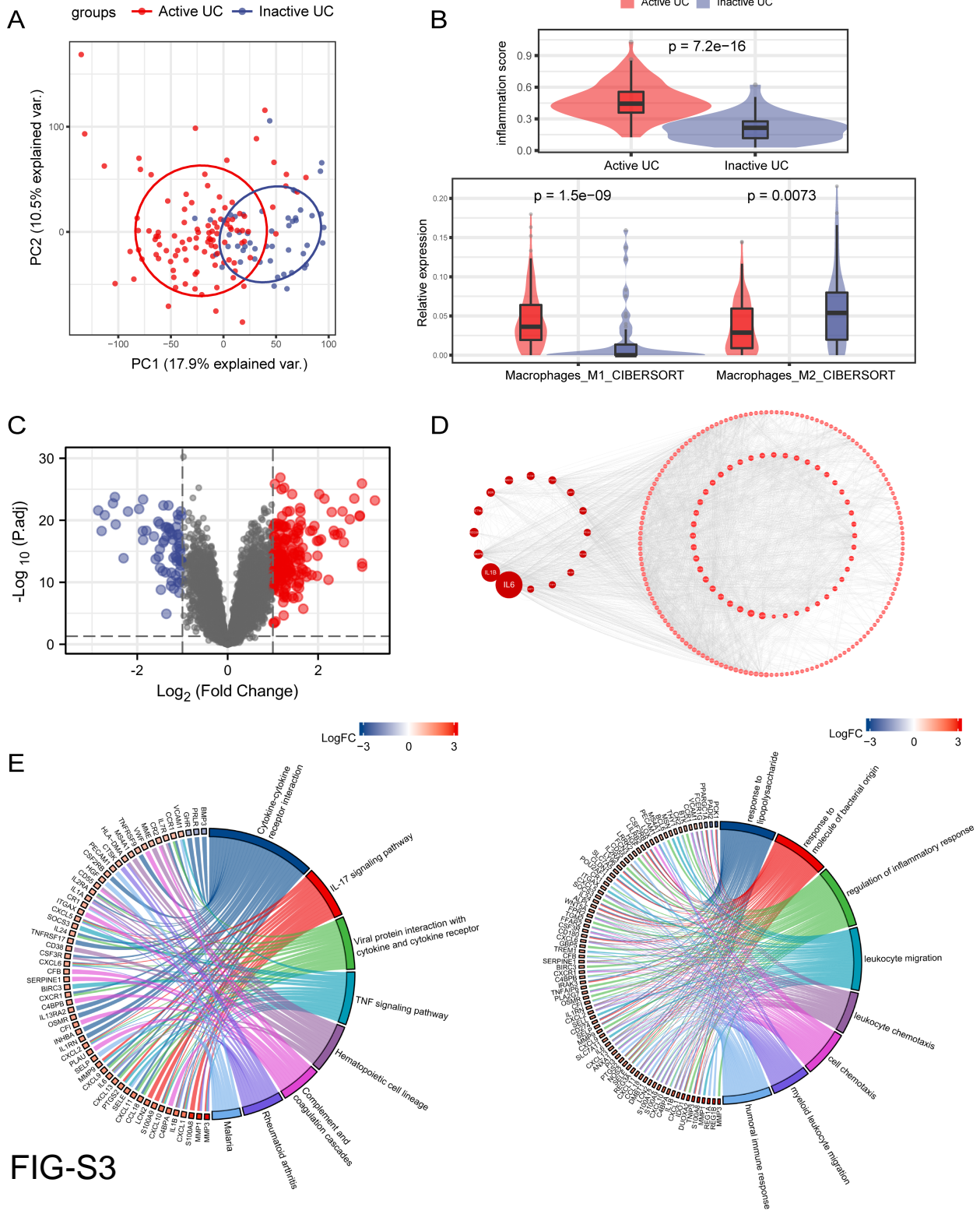

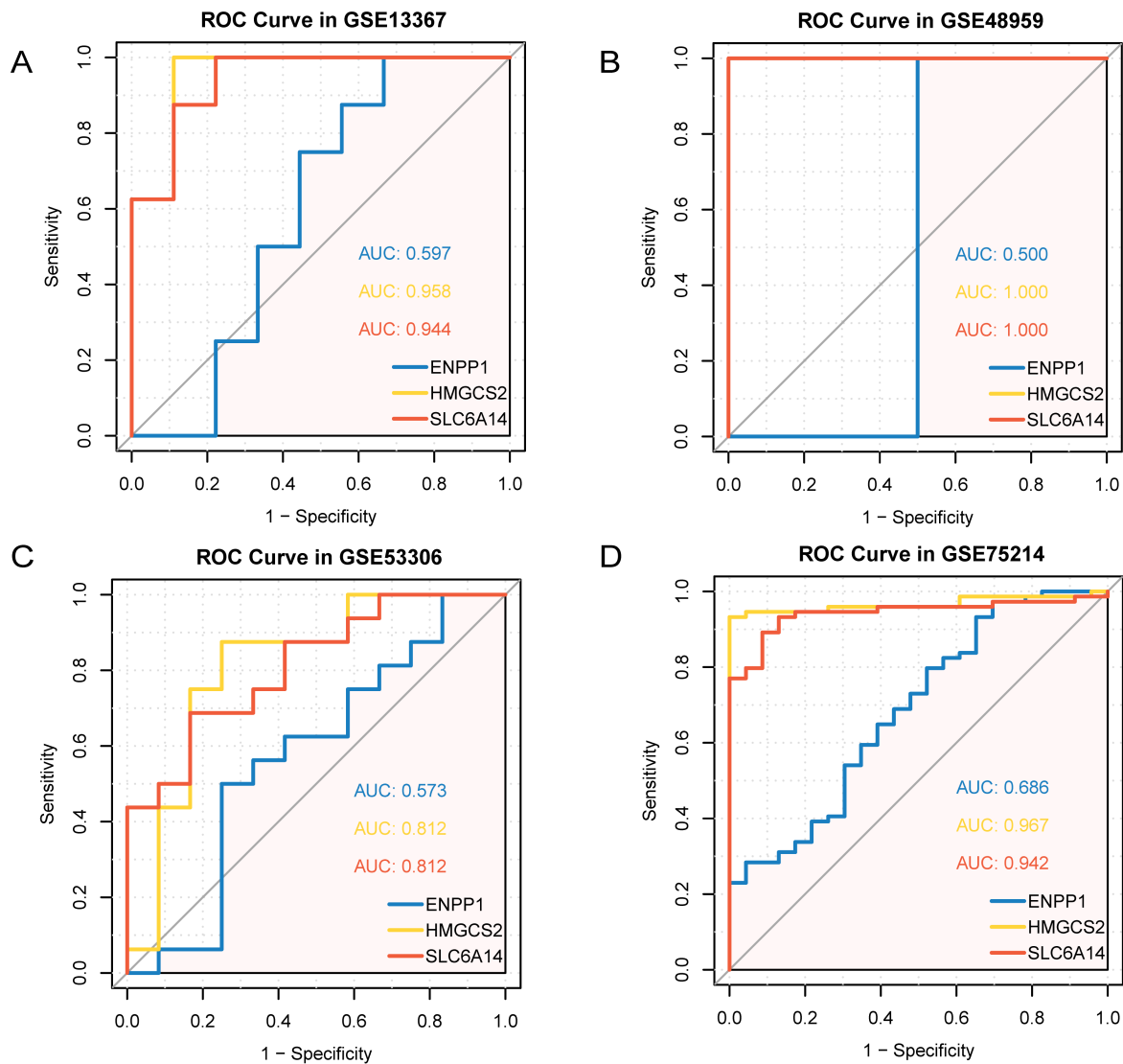

FIG-S4

A

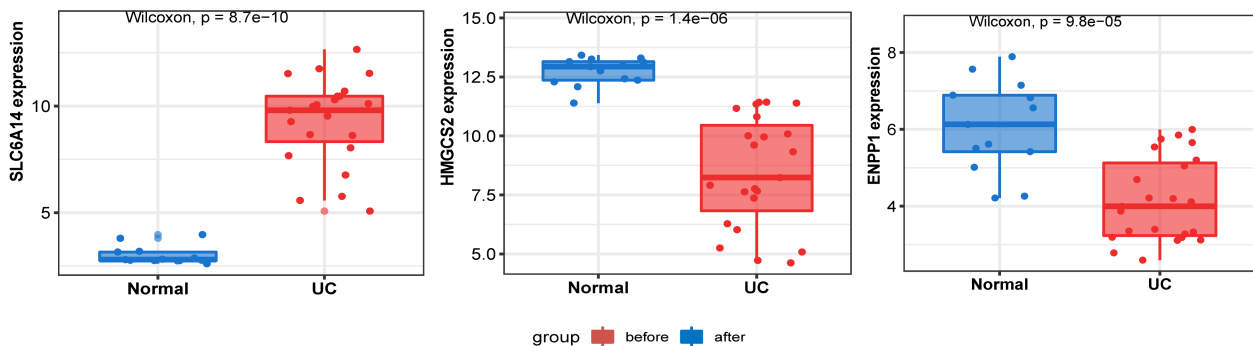

B

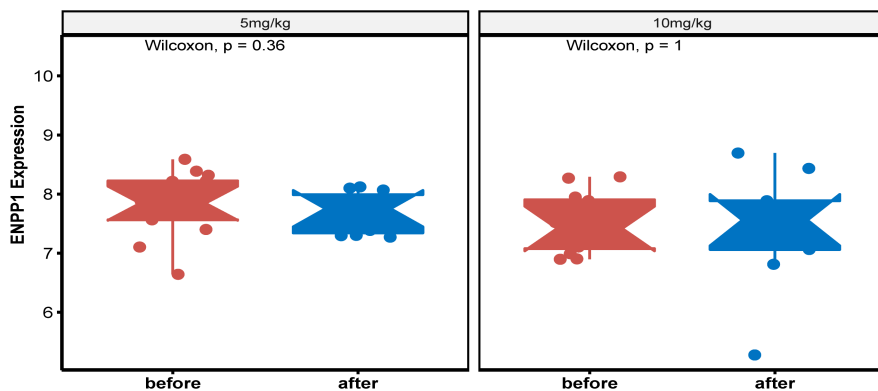

C

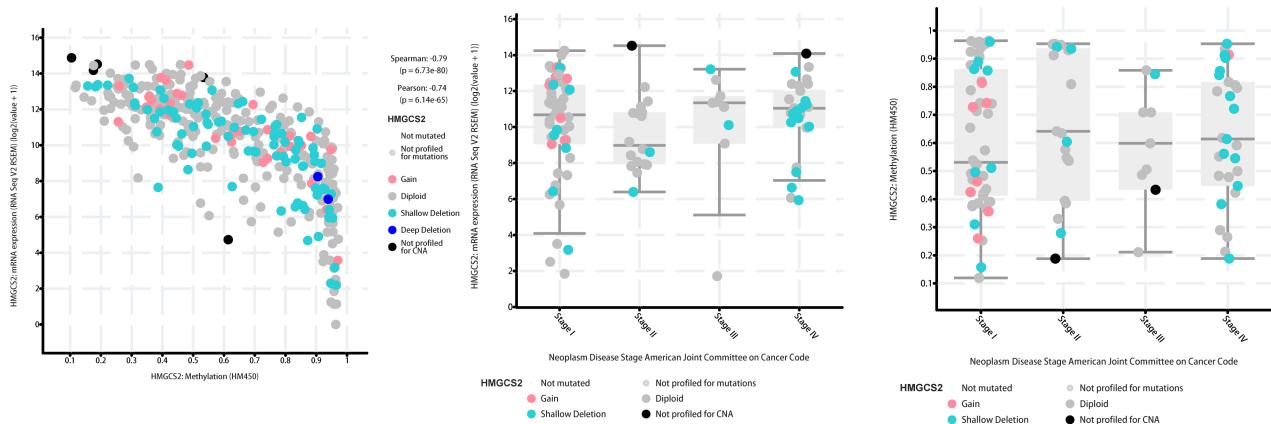

FIG-S5

Supplement: Supplementary 2 — Figure S1: integration of datasets batch correction and consensus clustering matrixes of UC patients. Figure S2: soft thresholding power and scale-free network validation in GSE36807 and GSE87466. Figure S3: differentially expressed genes between active and quiescent UC. Figure S4: diagnostic value of candidate diagnostic markers in identifying active UC. Figure S5: expression of candidate markers in the UC cohort of the GSE38713 dataset and in the TCGA CRC cohort with different tumor stages. [file 4373840.f2.pdf]
